# A thermodynamics-integrated physics-guided neural network for soil temperature forecasting

**DOI:** 10.1038/s41598-026-50274-y

**Published:** 2026-05-11

**Authors:** Shengyi Wang, Jinlong Zhu

**Affiliations:** College of Computer Science and Technology, Changchun Normal University, Changchun, 130031 China

**Keywords:** Soil temperature forecasting, Dissipation constraints, Thermodynamic potential, Physics-guided loss, Physics-informed neural networks, LSTM, Climate sciences, Environmental sciences, Hydrology, Mathematics and computing

## Abstract

Soil temperature forecasting plays a key role in agriculture, hydrology, and climate modeling; however, existing deep learning models often show degraded performance in long-term prediction due to error accumulation, insufficient physical interpretability, and limited spatial generalization. To overcome these limitations, this study proposes a Thermodynamic-Enhanced Physics-Informed Neural Network (TE-PINN), a forecasting framework based on an LSTM backbone that integrates domain-specific physical knowledge through the Latent Thermodynamic Potential Inference (LTPI) and Multi-Pathway Physics-Guided Loss Integration (MPPGLI) modules. LTPI applies free-energy principles and dissipation constraints to characterize internal thermal dynamics, addressing the difficulty of LSTM in capturing long-range temporal dependencies. MPPGLI provides a multi-path physics-guided loss formulation that effectively narrows the discrepancy between predictions and observations, improving robustness. TE-PINN exhibits slower performance degradation across multi-day forecast horizons and maintains stable predictive behavior across datasets from different latitudes. In comparison with both shallow and deep baseline models, the results indicate that introducing thermodynamic priors substantially improves the accuracy and physical consistency of soil temperature forecasting.

## Introduction

Soil temperature governs heat and moisture exchange at the land–atmosphere interface and regulates processes such as crop growth, soil biogeochemistry, permafrost dynamics, and hydrological extremes^1–3^. It co-varies with soil moisture and radiation, thereby modulating key feedbacks in energy and water cycles. Accurate soil temperature prediction is therefore fundamental for agriculture, hydrology, and climate modeling^4^.

Traditionally, data-driven models such as Recurrent Neural Networks (RNNs)^5,6^, Long Short-Term Memory (LSTM) networks^7,8^, and convolutional architectures (i.e., ConvLSTM) have been widely applied to capture temporal dependencies in environmental variables^9–11^. These models can approximate complex input-output mappings based on historical time series^12–14^, but they often lack the ability to follow physical constraints or generalize to unknown climate or geographical environments^15–17^, especially when dealing with soil temperature prediction. On one hand, purely data-driven models (e.g., LSTM-based architectures^18^) can effectively capture short-term nonlinear temporal dependencies but tend to accumulate errors during forecasting, especially when extrapolating beyond the training distribution. On the other hand, strongly enforced physical constraints–such as heat conduction equations–may overly restrict the hypothesis space of neural networks, limiting their ability to adapt to heterogeneous soil conditions and data noise. Although physics-guided models attempt to integrate physical knowledge into neural networks, they suffer from noise sensitivity, error accumulation, and a lack of robust depth-dependent representation^19^. Balancing the flexibility of data-driven approaches with the enforcement of physical constraints remains a significant challenge.

To overcome these limitations, physics-informed neural networks (PINNs) have emerged as a promising paradigm in environmental and geophysical modeling^20,21^. By embedding partial differential equations (PDEs) into loss functions, PINNs constrain the solution space of neural networks to remain consistent with known physical processes^22,23^. Recent studies demonstrate their effectiveness in forward and inverse problems such as contaminant transport^24^, groundwater flow^25^, and thermal diffusion^26,27^, showing improved accuracy and interpretability over purely data-driven approaches. For example, multi-fidelity physics-constrained neural networks integrate observational data with physical models to improve learning efficiency and predictive accuracy across different fidelity levels. Similarly, encoder–decoder neural network architectures have been developed for latent data assimilation in dynamical systems, where physical constraints are incorporated into the training objective to regularize the prediction of system states over time^28,29^. These approaches highlight the potential of combining sequential deep learning architectures with physics-based regularization for modeling complex temporal processes. However, most existing PINN frameworks are designed for synthetic or low-dimensional datasets and require explicit spatiotemporal boundary conditions, which limits their predictive capability in real-world applications. This is a complex problem because vertical heat diffusion, moisture coupling, and seasonal boundary forcing act on different spatial and temporal scales. Balancing these competing objectives within existing physics-guided soil temperature prediction frameworks remains an unresolved challenge.

In parallel, hybrid physics-guided deep learning models have been proposed as a more flexible alternative. By integrating domain knowledge with machine learning, these models aim to improve robustness and generalization. For instance, physics-guided LSTM frameworks and energy-constrained neural networks have been applied in hydrology and climate science^30,31^. They typically leverage known conservation laws (i.e., energy balance) or physically derived constraints (i.e., flux limits and state variable bounds) to regularize the learning process^32–34^. Many existing physics-guided approaches adopt single-pathway constraints or isolated penalties, which may over-regularize certain processes while leaving others unconstrained. Similar challenges of model imbalance and representation bias have also been observed in other machine learning domains, where specialized architectures are designed to address data and task imbalance^35^.

This work addresses these gaps by introducing a thermodynamic-enhanced, physics-informed prediction framework (TE-PINN) for soil temperature. In long-horizon autoregressive forecasting, small one-step prediction errors can accumulate during iterative rollouts, gradually causing the predicted trajectory to drift away from physically plausible temperature evolution. To address this challenge, the proposed TE-PINN framework introduces thermodynamically guided latent dynamics and diffusion-consistency constraints to regulate the temporal evolution of hidden states. Specifically, TE-PINN constrains the learned latent representation through thermodynamic admissibility and energy-dissipation principles, while simultaneously enforcing diffusion-consistent temperature evolution through physics-guided regularization. By stabilizing the evolution of latent states and restricting physically implausible trajectories, the framework effectively mitigates error accumulation during multi-step forecasting and improves the physical consistency of long-term soil temperature predictions. Built on an LSTM encoder, TE-PINN contributes two tightly coupled modules, namely Latent Thermodynamic Potential Inference (LTPI) and Multi-Pathway Physics-Guided Loss Integration (MPPGLI). Based on the efficient capability of LTPI to capture long-term temperature trends, MPPGLI forms a balanced multi-objective regularizer in which thermodynamic consistency, diffusion dynamics, and data fidelity are jointly optimized. This adaptive integration prevents dominance of any single constraint, thereby reducing variance without inflating bias. Practically, the multiple pathway links internal thermodynamic evolution to externally observable transport, yielding slower error accumulation over long-term and improved depth-wise robustness. The main contributions of this study are summarized as follows.

- A thermodynamic-enhanced physics-informed prediction framework (TE-PINN) is introduced, which embeds physically meaningful priors into the LSTM architecture, compensating for the poor performance of the original LSTM in long-term dependency prediction, thereby improving the robustness and stability of long-term forecasting.
- A Latent Thermodynamic Potential Inference (LTPI) module is constructed, which combines free energy and dissipation constraints to simulate the distributional variation of internal soil temperature, optimizing the noise diffusion problem during prediction over time. This enables the model to better capture long-term dependencies and regulate latent state evolution, thereby limiting error accumulation during forecasting.
- A Multi-Pathway Physics-Guided Loss Integration (MPPGLI) module is designed, which integrates PDE residuals, auxiliary latent supervision, and prediction errors into a composite multi-objective regularizer, improving the model’s fitting ability while enforcing trajectory-level physical consistency, thereby reducing physically inconsistent evolution in long-term forecasts.
- Validated on the representative LandBench dataset, experimental results show that TE-PINN overall outperforms mainstream shallow and deep baseline methods, while maintaining high robustness in multi-day forecasts and superior generalization ability across different climatic zones.

## Thermodynamic foundations and dataset description

This section establishes the theoretical and data-driven foundations of the proposed framework. We introduce key thermodynamic principles, including energy conservation laws, heat conduction equations, and free energy-based dissipation mechanisms, which form the physical basis of our model design. Then, we describe the LandBench dataset and preprocessing pipeline, detailing the static and dynamic variables used for soil temperature forecasting.

### Thermodynamic principles for soil heat and moisture transport

#### Macroscopic energy conservation and soil heat transfer

According to the first law of thermodynamics applied to variably saturated porous soils^36^, the local energy balance can be expressed as the Eq. (1).

1 $$\begin{aligned} \rho c\left( \theta \right) \frac{\partial T}{\partial t}=\nabla \cdot \left( k\left( \theta \right) \nabla T\right) +Q \end{aligned}$$

where *T* is the soil temperature, $\rho$ is the soil bulk density, $c(\theta )$ is the specific heat capacity that depends on the water content $\theta$, $k(\theta )$ is the thermal conductivity, which typically increases with soil water content as water-filled pores provide more efficient heat conduction pathways than air-filled pores, *Q* represents heat sources or sinks (e.g., solar radiation, root heat release, etc.).

In unsaturated soils, complex heat-water-air interactions cause thermal transport to be driven not only by temperature gradients but also significantly influenced by water phase states^37,38^. Experimental evidence demonstrates increased soil water content facilitates liquid water flow. This water forms preferential paths through soil pore networks. These preferential paths create water bridges. The resulting water bridges enhance thermal conduction^39^. This leads to a nonlinear and strongly increasing trend in $k(\theta )$, which must be accurately modeled through parameterization or implicitly captured by neural network structures.

In this study, soil temperature *T* is selected as the primary predictive variable. The spatial temperature gradient term and the source term *Q* are incorporated into the physical modeling process. External forcing processes represented by *Q* are accounted for through the meteorological input variables that drive the temperature evolution learned by the neural network. The residual of the above PDE serves as a supervisory signal embedded into the neural network via the physics-informed loss term $L_{pde}$, providing a physically grounded mechanism to enhance model interpretability.

Given that soil temperature observations are available only at several vertical depths and that horizontal heat fluxes within each grid cell are relatively small compared with vertical conductive fluxes, the heat transfer process is approximated using a one-dimensional vertical diffusion formulation along the soil column. This soil-column assumption is widely adopted in land surface modeling to represent the dominant vertical heat transport mechanism within each grid cell. In regions where lateral heat or moisture transport may be stronger, the one-dimensional diffusion constraint still captures the dominant vertical heat transfer process, while the data-driven components of the network can partially compensate for unresolved horizontal processes through learned spatiotemporal correlations. To ensure consistency between the continuous control equations and the discrete depth observation results, the vertical diffusion term is reformulated in discrete form. For soil temperature measured at layered depths $z_1, z_2, z_3$, the second-order derivative along the vertical direction is approximated using a central finite-difference scheme in Eq. (2).

2 $$\begin{aligned} \left. \frac{\partial ^2 T}{\partial z^2}\right| _{z_2} \approx \frac{T(z_3) - 2T(z_2) + T(z_1)}{(\Delta z)^2} \end{aligned}$$

where $\Delta z$ denotes the effective depth spacing between adjacent soil layers. Substituting this approximation into Eq. (1) yields a discrete residual form that preserves the thermodynamic structure of the energy conservation law while making it directly computable within the neural network training framework. In this study, the soil layers are not uniformly spaced in the vertical direction. To maintain a simple and stable discretization scheme, $\Delta z$ is defined as an effective depth spacing between adjacent observation layers, computed based on the representative distances between layer centers. This approximation enables the use of a standard central finite-difference formulation while preserving the dominant vertical heat diffusion characteristics. Although the non-uniformity of layer thickness may introduce approximation errors in the second-order derivative, these effects are mitigated by the data-driven components of the model and the physics-guided loss constraints during training. As a result, the discretized PDE residual remains a reliable surrogate for enforcing diffusion-consistent temperature evolution.

In practice, the resulting residual is incorporated into the physics-guided loss function of the neural network, allowing the model to enforce diffusion-consistent temperature evolution during training. The detailed formulation of residual-based loss will be introduced in Section subsection. It is worth noting that the specific functional forms of the free-energy potential and dissipation losses are not uniquely restricted in the proposed framework. In this study, the free-energy function is parameterized using a neural network and the dissipation term is derived through its thermodynamic conjugate variables, which provides a convenient and differentiable formulation for model training. More importantly, this formulation enforces thermodynamic consistency by linking latent-state evolution to an energy-based potential and its associated dissipation mechanism. This structure constrains the temporal dynamics of the internal variables and prevents physically inconsistent trajectories that commonly arise in purely data-driven sequence models. Compared with heuristic physical penalties that directly enforce PDE residuals, the energy–dissipation formulation provides a thermodynamically consistent mechanism to regulate latent-state evolution and improve long-horizon stability.

#### Energy coupling and free energy representation

In unsaturated soil systems, the fundamental driving forces of energy and moisture migration can be described by the Helmholtz free energy function per unit mass^40^, as shown in Eq. (3).

3 $$\begin{aligned} \psi =\psi \left( T,\theta \right) \end{aligned}$$

where *T* denotes the soil temperature and $\theta$ represents the volumetric water content. Based on this formulation, two key physical quantities can be derived, namely the entropy density (indicating the degree of disorder) and the soil water potential (capillary suction)^41^. These are presented in Eq. (4) and (5), respectively.

4 $$\begin{aligned}&s = - \frac{\partial \psi }{\partial T} \end{aligned}$$

5 $$\begin{aligned}&\mu = \frac{\partial \psi }{\partial \theta } \end{aligned}$$

According to the Clausius–Duhem inequality from irreversible thermodynamics^42^, the local dissipation rate *D* must satisfy the condition shown in Eq. (6).

6 $$\begin{aligned} D = \sigma : \dot{\varepsilon } - \rho \frac{d\psi }{dt} + T \rho \frac{ds}{dt} \ge 0 \end{aligned}$$

Under the isothermal assumption, this reduces to Eq. (7).

7 $$\begin{aligned} D = - \frac{\partial \psi }{\partial \theta } \cdot \frac{d\theta }{dt} \ge 0 \end{aligned}$$

This relation indicates that water migration in soil leads to a decrease in the system’s total free energy, consistent with the irreversibility of natural dissipative processes. To characterize the thermodynamic state of the system, we adopt a free energy density function as defined in Eq. (8).

8 $$\begin{aligned} \psi = \psi (F, z_1, z_2, \ldots , z_m) \end{aligned}$$

where *F* denotes the deformation gradient tensor (which reduces to the scalar temperature *T* in this study), and $\{z_1, z_2, \ldots , z_m\}$ represent internal state variables that capture latent thermal behaviors, learned from input time series via an LSTM encoder^43,44^. In this study, the free energy function $\psi$ is parameterized as a differentiable neural mapping that takes the predicted soil temperature *T* and the latent internal variables $\textbf{z}_t = (z_1, z_2, \dots , z_m)$ as inputs in Eq. (9).

9 $$\begin{aligned} \psi = \mathcal {N}_\psi (T, \textbf{z}_t; \boldsymbol{\theta }_\psi ) \end{aligned}$$

where $\mathcal {N}_\psi$ denotes a feedforward neural network with learnable parameters $\boldsymbol{\theta }_\psi$.

Here, *T* represents the macroscopic thermal state, while the latent variables $\textbf{z}_t$ encode sub-grid thermodynamic effects associated with soil moisture redistribution and internal thermal memory. This parameterization ensures that $\psi$ remains continuously differentiable with respect to *T* and $\textbf{z}_t$, enabling consistent computation of thermodynamic conjugate quantities and dissipation constraints.

Applying the Clausius–Duhem inequality again under isothermal conditions, the simplified dissipation condition is denoted as the Eq. (10).

10 $$\begin{aligned} d = P : \dot{F} - \dot{\psi } \ge 0 \end{aligned}$$

The total derivative of $\psi$ expands as

11 $$\begin{aligned} \dot{\psi } = \frac{\partial \psi }{\partial F} : \dot{F} + \sum _{a=1}^{m} \frac{\partial \psi }{\partial z_a} \cdot \dot{z}_a \end{aligned}$$

Substituting into the inequality gives

12 $$\begin{aligned} d = \left( P - \frac{\partial \psi }{\partial F}\right) : \dot{F} - \sum _{a=1}^{m} \frac{\partial \psi }{\partial z_a} \cdot \dot{z}_a \ge 0 \end{aligned}$$

To ensure this inequality holds for arbitrary $\dot{F}$, the constitutive relations must satisfy

13 $$\begin{aligned} P = \frac{\partial \psi }{\partial F}, \quad D = - \sum _{a=1}^{m} \frac{\partial \psi }{\partial z_a} \cdot \dot{z}_a \ge 0 \end{aligned}$$

Here, *P* is the first Piola-Kirchhoff stress, and *D* is the internal dissipation rate, which must remain non-negative. The variables $z_a$ capture latent thermal dynamics and indirectly encode the internal state of the soil. In our model, the internal variable $z_t \in \mathbb {R}^d$ is learned through an LSTM encoder, and the free energy function $\psi (z, C)$ is produced by a feed-forward neural subnetwork. The system’s dissipation rate $D_t$ is defined as the inner product between the thermodynamic force and the latent variable evolution^45^.

14 $$\begin{aligned} \tau _t = \frac{\partial \psi }{\partial z_t}, \quad \Delta z_t = z_t - z_{t-1}, \quad D_t = - \tau _t \cdot \Delta z_t \end{aligned}$$

To penalize violations of the second law of thermodynamics $(D_t < 0)$, we introduce the dissipation loss $L_{\text {diss}}$ as follows.

15 $$\begin{aligned} L_{\text {diss}} = \frac{1}{\tau }\sum _{t=1}^{\tau } \text {ReLU}(-D_t) \end{aligned}$$

This term softly constrains the model to maintain thermodynamic irreversibility throughout prediction. To further promote physically plausible energy evolution, we define the energy accumulation loss $L_{\text {energy}}$ to penalize violations of the non-negative dissipation condition in free energy evolution.

16 $$\begin{aligned} L_{\text {energy}} = \frac{1}{\tau }\sum _{t=1}^{\tau } \text {ReLU}(\psi _t - \psi _{\text {init}}) \end{aligned}$$

where $\psi _{\text {init}}$ is the free energy at the initial time step. This loss enhances the model’s ability to learn energy-dissipative and stable dynamics. It is important to note that the ReLU operator acts as a one-sided penalty, which is only activated when the free energy evolution violates the thermodynamic consistency condition. Therefore, physically admissible energy fluctuations are preserved, while only non-physical energy-increasing behaviors are penalized. This design provides a soft constraint mechanism that balances physical consistency and model flexibility.

### Data Description and Preprocessing

This work utilizes the LandBench 1.0 dataset^46^, an open-access benchmark specifically designed for Land Surface Variable (LSV) prediction. It integrates multiple authoritative sources, including ERA5-Land reanalysis, MODIS remote sensing products, and SoilGrids, into a harmonized framework with physics-consistent interpolation and region-wise normalization. All variables are resampled to a unified spatial grid (180 $\times$ 360) and daily temporal resolution before being used as model inputs.

**Figure 1:**
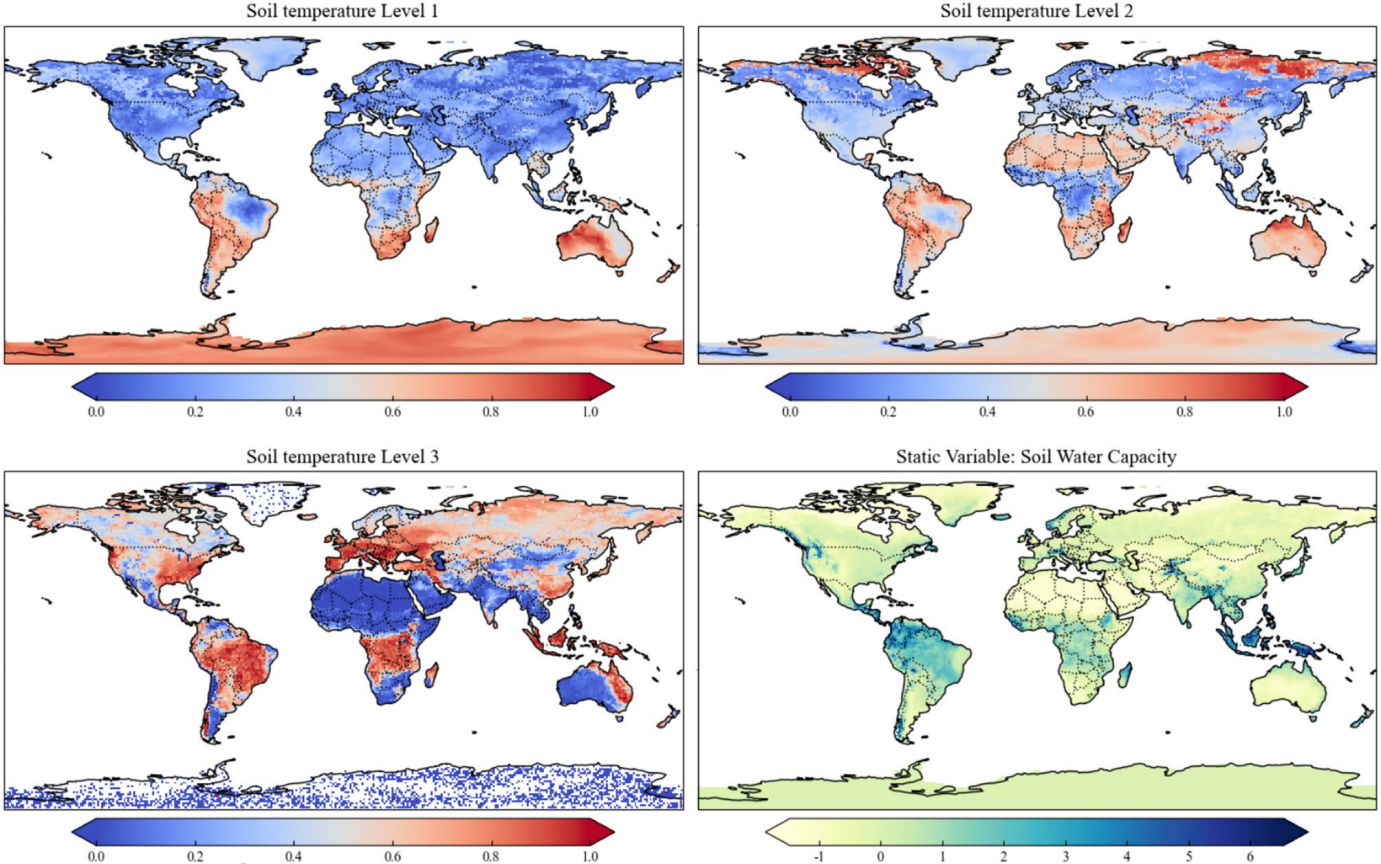
Visualizations of global distributions of selected dynamic and static variables.

We defined a supervised learning task for soil temperature. Input features fall into two categories. First, dynamic variables comprise daily time-series data recording soil moisture and multi-layer soil temperatures. Additional dynamic variables include total runoff and surface sensible heat flux (nine variables in total). Second, static variables represent soil water capacity (SWC), which characterizes the soil’s ability to retain water and influences infiltration and runoff processes. In this study, soil water capacity is adopted as the primary static descriptor because it directly influences soil moisture availability and thermal properties. This design allows us to isolate the contribution of the proposed physics-guided learning mechanisms and thermodynamic constraints. While additional static factors such as topography may also affect regional temperature patterns, especially in complex terrain, part of these effects are already reflected in the ERA5-Land meteorological forcing variables, which incorporate elevation-dependent atmospheric processes. For visualization analysis, we randomly selected three dynamic variables and one static variable to illustrate the overall distribution of soil temperature, with details shown in Fig. 1. All variables underwent unified preprocessing, including spatial cropping (180$\times$360 grid), gap filling, and normalization. Dynamic inputs are organized into temporal sliding windows for training and testing. Static inputs are broadcasted across time steps. In contrast to some previous studies that incorporate static environmental variables such as topography or soil type, the present work focuses primarily on dynamic meteorological forcing variables. This design choice aims to isolate the contribution of physics-guided learning mechanisms and thermodynamic constraints in improving soil temperature prediction. The soil temperature evolution in this study follows the classical one-dimensional heat diffusion equation $\partial T/\partial t = \alpha \partial ^2 T/\partial z^2$. The initial condition is represented by the historical temperature sequence used as model input, which approximates the initial soil temperature profile $T(z,t_0)$. At the soil surface, the observed surface temperature provides the upper boundary condition $T(0,t)=T_s(t)$. For deeper soil layers, the relatively slow thermal variation allows the lower boundary to be approximated by a zero-gradient condition $\partial T/\partial z(z_b,t)=0$. Under these initial and boundary conditions, the governing diffusion equation is enforced through the PDE residual constraint embedded in the training loss. Final inputs are stored in *.npy and *.memmap formats to support efficient large-scale training. The detailed model configuration and hyperparameter settings are presented in the Numerical Experiments section.

**Figure 2:**
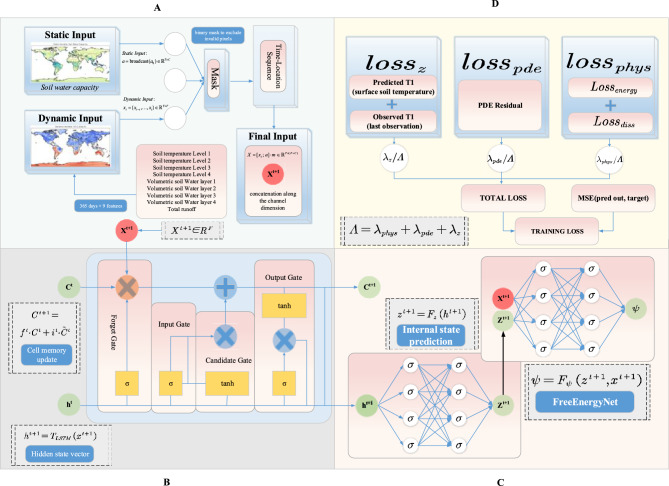
Conceptual architecture of the proposed TE-PINN model. The network is composed of four interlinked modules: (**A**) spatiotemporal input construction, where dynamic observations and static attributes are preprocessed and aligned; (**B**) temporal encoding via stacked LSTM units to capture sequential dependencies; (**C**) thermodynamic potential inference based on latent state vectors; and (**D**) multi-pathway physics-guided loss design integrating PDE residuals, free energy dissipation, and latent supervision. The architecture is designed to maintain physical consistency during training while enabling spatial generalization and long-range forecasting capability.

## Methods

To address the inherent complexity of coupled soil heat-moisture dynamics, we propose a Thermodynamic-Enhanced Physics-Informed Neural Network (TE-PINN). This hybrid framework integrates data-driven temporal modeling via LSTM networks with thermodynamic priors and partial differential equation (PDE)-based physical constraints, aiming to ensure physically consistent soil temperature prediction. TE-PINN unifies dynamic observations, static surface parameters, and latent thermodynamic structures into an end-to-end forecasting pipeline that simultaneously enforces energy conservation and maintains flexible spatiotemporal generalization. This section first introduces the overall architecture and training logic of the TE-PINN, outlining its modular structure and progressive optimization process. It then provides a detailed analysis of the four key modules (A-D), each of which will be examined individually in the following sections.

### TE-PINN architecture

The proposed TE-PINN model is designed as a modular and interpretable architecture that embeds physical consistency directly into the learning process. Instead of treating soil temperature forecasting as a purely black-box sequence-to-sequence problem, the network explicitly incorporates prior knowledge of energy transfer mechanisms, multi-layer interactions, and latent thermodynamic processes. This is achieved by structuring the model into four functional components that correspond to distinct stages of spatiotemporal data assimilation, dynamical encoding, physics-informed reasoning, and supervised prediction.

The complete computational pipeline of TE-PINN is illustrated in Fig. 2, while the corresponding high-level algorithmic flow is summarized in Algorithm 1. The architecture consists of four interdependent modules (denoted as A-D), each responsible for a key functional component of the network, including input sequence generation and spatial broadcasting (Module A), temporal encoding via stacked LSTM cells (Module B), thermodynamic potential construction via latent feature projection (Module C), and physics-informed loss formulation incorporating energy and diffusion residuals (Module D).

A key motivation of the TE-PINN framework is to mitigate error accumulation in long-horizon autoregressive forecasting. In conventional sequence models, small one-step prediction errors may propagate through the hidden states during iterative rollouts, causing the predicted trajectory to gradually drift away from physically consistent temperature evolution. To address this issue, TE-PINN introduces physics-guided constraints that regulate the temporal evolution of latent representations. The thermodynamic supervision module constrains the latent potential and its temporal dynamics through energy-dissipation principles, while the diffusion-consistency constraint encourages the predicted temperature field to follow the governing heat conduction behavior along the soil column. By jointly guiding latent-state evolution and temperature diffusion dynamics, the proposed framework stabilizes long-term forecasting trajectories and reduces the accumulation of prediction errors during multi-step prediction.

To ensure a systematic and physically grounded training process, the computational workflow of TE-PINN is summarized in Algorithm 1. The algorithm outlines the full optimization loop across training epochs, from data ingestion and latent encoding to thermodynamic supervision and multi-objective loss aggregation. Each functional component aligns with one of the architectural blocks shown in Fig. 2, allowing the model to progressively enforce physical consistency alongside prediction accuracy. Notably, thermodynamic supervision is achieved via a latent potential $\psi$, its time derivative $\dot{\psi }$, and a dissipation-constrained flux term *v*, all of which are incorporated into the loss function design.

**Algorithm 1:**
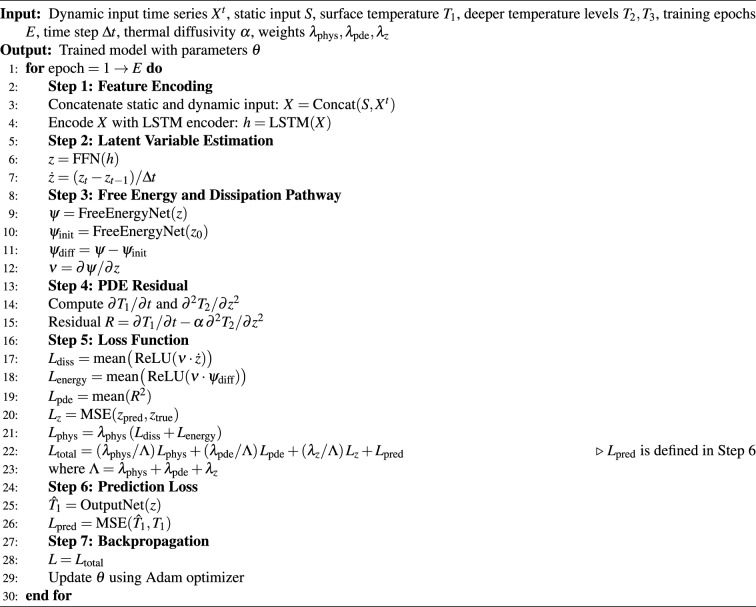
Training procedure of TE-PINN

The architecture of TE-PINN facilitates a systematic evaluation of individual components in terms of physical consistency and predictive robustness. During the previous section, we dissect each module in detail, beginning with the data preprocessing and input generation process (Module A), and proceeding sequentially through temporal modeling (Module B), thermodynamic projection (Module C), and physics-guided supervision (Module D).

### Spatiotemporal input encoding and variable construction

**Figure 3:**
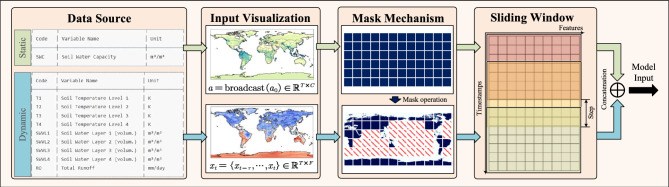
Spatiotemporal input encoding pipeline of TE-PINN. The process corresponds to Module A in the overall architecture in the Fig. 2 and is formally implemented as Step 1 in Algorithm 1.

In order to effectively capture soil thermodynamic processes across both spatial and temporal scales, we designed a structured preprocessing pipeline as the first step of the TE-PINN framework. This pipeline encodes heterogeneous inputs, comprising static surface properties and dynamic environmental variables into a unified learnable representation as illustrated in Fig. 3. It corresponds to the input construction process in Module A of the overall architecture in the top left of Fig. 2, and is implemented as Step 1 in Algorithm 1, where model-ready tensors are constructed for downstream temporal encoding.

The static input *S* includes spatially invariant but location-specific properties such as Soil Water Capacity (SWC), which governs infiltration and water retention behavior. This variable is broadcast across all time steps to match the temporal dimension of the dynamic inputs. The dynamic input $X^t$, on the other hand, comprises nine time-varying surface variables derived from the LandBench dataset, including multi-depth soil temperature levels (T1-T3), volumetric soil water content (SWVL1-SWVL4), and total runoff. Each dynamic input is recorded daily and encoded as a multivariate temporal sequence. To ensure spatial consistency and avoid noise from invalid regions (e.g., oceans or missing data), a land-sea mask is applied uniformly across both static and dynamic layers. This mask operation filters out irrelevant grid cells prior to concatenation. Subsequently, all valid inputs are concatenated along the feature dimension, as defined in Eq. (17) . The broadcast-and-concatenation strategy is adopted to fuse static and dynamic variables for two reasons. First, the static variables represent location-specific but temporally invariant soil properties, which mainly serve as conditioning factors for the temporal evolution of dynamic variables. Broadcasting these variables across time steps allows the LSTM encoder to incorporate spatial context at each temporal state without disrupting sequential modeling. Second, this lightweight fusion mechanism preserves a stable feature structure for long temporal sequences while avoiding the additional parameters introduced by more complex interaction modules. As discussed later in Section subsection, empirical experiments further confirm that introducing additional attention-based interaction does not improve predictive performance for this task.

17 $$\begin{aligned} X=Concat\left( S,X^t\right) \end{aligned}$$

where $X\in R^{T\times P}$ denotes the input of the proposed model. Here *T* is the number of time steps and *P* the feature dimensionality after concatenation. As illustrated in Fig. 3, the input pipeline consists of four stages: data source preparation, input visualization, mask mechanism, and sliding-window segmentation. The resulting sequence is segmented into overlapping windows to generate sample blocks for supervised training, enabling the model to capture temporal dependencies and regional variability simultaneously. The constructed input tensor is then passed into the LSTM encoder in Section subsection for sequential modeling.

### LSTM encoder-based temporal dynamics modeling

To capture the temporal dependencies underlying the soil thermodynamic process, we employ a sequential encoding module based on LSTM networks. As shown in Module B of Fig. 2, this module processes the model input *X* constructed in Section subsection, transforming it into a series of latent state vectors that reflect the system’s historical behavior. This step corresponds to Steps 2-3 in Algorithm 1. The *X* is first passed through a stacked two-layer LSTM, which encodes both short- and long-range interactions across time. The resulting hidden state sequence $h = \operatorname {LSTM}(X)$ is then projected into a latent thermodynamic state $z \in \mathbb {R}^{d}$ through a feed-forward network, as shown in Eq. (18). The latent variable *z* is interpreted as a thermodynamic state variable that characterizes the internal state of the soil system. Each dimension of *z* encodes physically meaningful information associated with key processes governing soil temperature evolution, including heat storage, thermal inertia, and moisture-modulated energy retention. In particular, *z* serves as an effective representation of the system’s thermal memory, capturing the cumulative influence of past environmental forcing and subsurface heat diffusion. This establishes a physically grounded correspondence between the latent state and underlying processes such as soil moisture effects and energy transport dynamics. Furthermore, the temporal evolution of *z* is constrained through subsequent thermodynamic potential modeling and physics-guided loss functions, ensuring that its dynamics remain consistent with energy conservation and dissipation principles. Consequently, *z* serves as a physically interpretable bridge between data-driven temporal encoding and thermodynamic modeling.

18 $$\begin{aligned} z = \operatorname {FFN}(h) \end{aligned}$$

To approximate the temporal evolution of this latent state, we compute the first-order time derivative, as expressed in Eq. (19).

19 $$\begin{aligned} \dot{z} = \frac{z_{t} - z_{t-1}}{\Delta t} \end{aligned}$$

This formulation allows the model to infer not only static representations, but also the dynamic transition behavior of physical quantities.

In the next step, this latent representation is used to parameterize the thermodynamic potential, serving as the foundation for downstream energy-based modeling in Section subsection. By structuring this temporal encoder as a separate, interpretable component, the network is able to isolate dynamic factors before introducing physical constraints.

### Latent thermodynamic potential inference

To incorporate thermodynamic interpretability into the network, we introduce a latent free energy modeling pathway named *FreeEnergyNet*, as shown in Module C of Fig. 2, corresponding to Step 3 in Algorithm 1. This module receives the latent representation *z* produced by the LSTM encoder. The vector *z* serves as an internal latent state of the system from which the thermodynamic free-energy potential $\psi$ is parameterized. Environmental forcing variables are first encoded by the LSTM encoder and therefore influence the inferred free-energy potential $\psi$ indirectly through the latent representation *z*, as shown in Eq. (20).

20 $$\begin{aligned} \psi = \operatorname {FreeEnergyNet}_{\psi }(z) \end{aligned}$$

In addition, the network estimates the energy deviation from the initial thermodynamic state, defined as in Eq. (21).

21 $$\begin{aligned} \psi _{\text {diff}} = \psi - \psi _{\text {init}} \end{aligned}$$

This scalar captures the temporal accumulation of system energy relative to an initial configuration, and serves as a basis for inferring dissipation dynamics. To characterize the internal driving force behind system evolution, we further compute the gradient of the free energy potential with respect to the latent variables, as expressed in Eq. (22).

22 $$\begin{aligned} \nu = \frac{\partial \psi }{\partial z} \end{aligned}$$

The thermodynamic potential function $\psi$ is parameterized as a differentiable neural mapping from the latent state to a scalar free-energy value. Owing to the use of smooth nonlinear activation functions (e.g., Softplus), $\psi$ remains continuously differentiable with respect to the latent variables, which enables the definition of thermodynamic force-like quantities through $\partial \psi / \partial z$. Within the proposed framework, thermodynamic consistency is primarily enforced through the energy–dissipation mechanism embedded in the loss function. In particular, the dissipation-related constraint $L_{\textrm{diss}}$ and energy-based constraint $L_{\textrm{energy}}$ regulate the temporal evolution of the latent variables, promoting non-negative dissipation behavior and preventing unphysical energy oscillations. This effectively constrains the local geometry of the learned energy landscape and stabilizes the system dynamics.In addition, the latent representation is implicitly bounded and regularized during training, which further contributes to shaping a smooth and stable energy surface. We note that the current implementation does not impose a strict architectural constraint to guarantee global convexity or homogeneity of $\psi$. Instead, these properties are approximated in a training-driven manner through smooth parameterization and physics-guided regularization, which has been found sufficient to ensure stable and physically admissible temperature evolution in practice.

This latent force $\nu$ provides a mechanistic interpretation of the system’s movement in the energy landscape and will be used in subsequent physics-based loss functions described in Section subsection to enforce energy-constrained behavior during training.

### Multi-pathway physics-guided loss integration

To align the model training process with underlying physical principles, we design a composite loss framework that integrates multiple physics-guided components. As illustrated in Module D of Fig. 2, this design includes three primary loss terms: prediction loss $L_{z}$, thermodynamic energy loss $L_{\text {phys}}$, and PDE-based residual loss $L_{\text {pde}}$. The corresponding weighting coefficients $\lambda _{\textrm{phys}}$, $\lambda _{\textrm{pde}}$, and $\lambda _{z}$ are defined in a normalized form, such that their relative contributions are controlled under a fixed total weight budget. Specifically, in the sensitivity analysis, one coefficient is swept while the remaining two are equally assigned from the residual portion, e.g., $\lambda _{\textrm{pde}}=\lambda _{z}=(1-\lambda _{\textrm{phys}})/2$, $\lambda _{\textrm{phys}}=\lambda _{z}=(1-\lambda _{\textrm{pde}})/2$, and $\lambda _{\textrm{phys}}=\lambda _{\textrm{pde}}=(1-\lambda _{z})/2$. This design follows the common weighted-sum formulation in multi-objective optimization, where nonnegative coefficients are normalized as a convex combination to enable stable and interpretable trade-off analysis. Under this normalization, the total supervision strength remains constant across different sweep settings, which avoids performance changes caused merely by global scale variation of the loss and makes the comparison among different pathways more fair and physically interpretable. This multi-pathway supervision strategy directly constrains the network from both observation-driven and physics-driven perspectives. The formulation corresponds to Steps 4–6 in Algorithm 1.

**Prediction supervision loss:** We first define an auxiliary supervision loss for the latent pathway. Specifically, the latent representation *z* is mapped through a shallow output layer to obtain an intermediate estimate of surface temperature $T_1$, which is then compared with the corresponding observed temperature. Where $\hat{T}_{1}^{\,z}$ denotes the surface temperature estimate decoded from the latent representation *z*, and $T_{1}^{\,\textrm{obs}}$ is the corresponding observed temperature. The associated loss is computed as in Eq. (23).

23 $$\begin{aligned} L_z = \textrm{MSE}(\hat{T}_{1}^{\,z}, T_{1}^{\,\textrm{obs}}) \end{aligned}$$

This term ensures that the latent representation remains consistent with surface temperature dynamics. **Thermodynamic consistency loss:** To enforce internal consistency of energy propagation, we incorporate the thermodynamic quantities obtained in Section subsection. Specifically, we penalize the dissipative deviation and the energy flux misalignment, as shown in Eq. (24) and (25).

24 $$\begin{aligned}&L_{\text {diss}} = \operatorname {mean}\!\big (\operatorname {ReLU}(\nu \cdot \dot{z})\big ) \end{aligned}$$

25 $$\begin{aligned}&L_{\text {energy}} = \operatorname {mean}\!\big (\operatorname {ReLU}(\nu \cdot \psi _{\text {diff}})\big ) \end{aligned}$$

The combined physics-based energy loss is expressed in Eq. (26).

26 $$\begin{aligned} L_{\text {phys}} = \lambda _{\text {phys}} (L_{\text {diss}} + L_{\text {energy}}) \end{aligned}$$

This regularization guides the model to conform to thermodynamic laws at the latent level. **PDE-based residual loss:** To further reflect physical dynamics along the soil column, we construct a residual term based on the one-dimensional heat conduction equation introduced in previous Section. Specifically, starting from the energy balance formulation in Eq. (1), and assuming dominant vertical heat diffusion, the governing equation can be written in the simplified form Eq. (27). Here, *z* denotes the vertical spatial coordinate representing soil depth in the physical heat diffusion equation, which is distinct from the latent variable *z* used in the neural network modules.

27 $$\begin{aligned} Residual_{\text {pde}} = \frac{\partial T_{1}}{\partial t} - \alpha \frac{\partial ^{2}T_{2}}{\partial z^{2}} \end{aligned}$$

The associated mean squared error is then used as a penalty, as shown in Eq. (28).

28 $$\begin{aligned} L_{\text {pde}} = \operatorname {mean}(Residual_{\text {pde}}^{2}) \end{aligned}$$

This term ensures vertical coherence between the observed surface and deeper soil temperature profiles. **Final Loss Formulation:** The overall training loss is defined as a weighted sum of the above components, as summarized in Eq. (29).

29 $$\begin{aligned} L_{\text {total}} = \frac{\lambda _{\text {phys}}}{\Lambda } L_{\text {phys}} + \frac{\lambda _{\text {pde}}}{\Lambda } L_{\text {pde}} + \frac{\lambda _{z}}{\Lambda } L_{z} \end{aligned}$$

where $\Lambda = \lambda _{\text {phys}} + \lambda _{\text {pde}} + \lambda _{z}$ is the normalization factor. This design ensures all loss components contribute proportionally while preserving interpretability and training stability. The weighting coefficients further allow flexible adjustment of the relative strength between data-driven fitting and physics-guided regularization. Finally, Step 7 in Algorithm 1 performs backpropagation and optimization to update all model parameters based on the composite training loss. To summarize, the proposed TE-PINN integrates heterogeneous inputs, temporal encoders, thermodynamic priors, and multi-pathway physics constraints into a cohesive deep learning framework. The architecture is fully differentiable and trained end-to-end, with each module contributing uniquely to temporal dynamics modeling and physical interpretability. This modular design enables flexible experimentation and ablation analysis across spatial, temporal, and physical dimensions, as detailed in the following section.

## Numerical experiments

### Experimental design and protocol

To rigorously evaluate the performance, stability, and physical interpretability of the proposed TE-PINN, we design a comprehensive suite of experiments that encompass temporal, architectural, and comparative perspectives. Specifically, the experiments cover four aspects: i) Architecture-Level Ablation; ii) Loss Weight-based Sensitivity Analysis; iii) Temporal Robustness Evaluation; and iv) Comparison with State-of-the-Art Models.

To prevent information leakage in time-series prediction, the dataset is partitioned chronologically rather than by random sampling. In the present implementation, the remaining pre-2020 observations are used for training. When validation is enabled, this pre-test period is further split chronologically, using the earlier 80% for training and the later 20% for validation. Furthermore, the sliding-window construction of input-target pairs is applied strictly within each temporal subset. Therefore, no training, validation, or testing sample shares temporal overlap across subset boundaries, ensuring a leakage-free and realistic forecasting evaluation setting.

Key model and training hyperparameters, including the LSTM encoder configuration (e.g., hidden size, number of layers, and dropout), are summarized in Table 1. These settings follow commonly adopted practices in LSTM-based sequence modeling^18,47^, where hidden dimensions on the order of $10^2$ and shallow encoder depths are typically used to balance temporal representation capability and training stability. In addition, moderate dropout regularization is introduced to mitigate overfitting in environmental time-series forecasting tasks. For the latent thermodynamic potential inference module, the free energy network is implemented with three layers. Specifically, the first layer projects the latent internal variable and scalar state variable into a thermodynamic feature space, the intermediate layer performs nonlinear energy transformation to characterize the local energy landscape, and the final layer outputs a scalar free-energy potential $\psi$. As summarized in Table 1, the free energy network uses an input dimension of 9, a hidden dimension of 30, and an output dimension of 1, with ReLU and Softplus activations adopted to provide nonlinear representation capacity and smooth energy mapping, respectively. This parameterization allows the module to serve as a compact thermodynamic projection block rather than a purely black-box predictor. For fair comparison, all baseline models and ablation variants are trained using the same core hyperparameter settings as the proposed TE-PINN, including the LSTM hidden dimension, number of encoder layers, training epochs, batch size, and optimizer configuration. No additional tuning is performed for individual baseline models.

**Table 1:** Key hyperparameter settings of the TE-PINN model and training configuration.

Parameter	Value
LSTM encoder layers	2
Hidden dimension	128
Dropout rate	0.15
Batch size	64
Learning rate	$1\times 10^{-3}$
Training epochs	100
Optimizer	Adam
Free energy network layers	3
Input dimension	9
Hidden dimension of free energy network	30
Output dimension	1
Activation functions	ReLU & Softplus

### Ablation studies

#### Architecture-level ablation

To systematically evaluate the contribution of each architectural component, we conduct a structured ablation analysis across four representative model configurations.

- **Full TE-PINN**: The complete architecture, integrating LSTM-based temporal encoders, Latent Thermodynamic Potential Inference, and Multi-Pathway Physics-Guided Loss Integration.
- **w/o LTPI**: In this variant, the supervision on latent thermodynamic states is disabled, including both the free energy loss and the free energy divergence loss.
- **w/o MPPGLI**: A further ablated version in which all externally imposed physical constraints are removed. Specifically, the heat-conduction PDE residual constraint and the auxiliary variable supervision are eliminated, leaving only the primary feed-forward prediction pathway, the LSTM backbone, and a minimal form of internal thermodynamic supervision.
- **LSTM**: A purely data-driven benchmark using an LSTM network without any physics-based or thermodynamic guidance.

**Table 2:** Architecture-level ablation results under different configurations. LSTM denotes the backbone sequence encoder; LTPI denotes the Latent Thermodynamic Potential Inference module; MPPGLI denotes the Multi-Pathway Physics-Guided Loss Integration module. Performance is evaluated using Median $R^{2}$, unbiased RMSE (ubRMSE), RMSE, and bias.

Model	LSTM	LTPI	MPPGLI	Median $R^{2}$	ubRMSE	$R^{2}$	RMSE	Bias
Model1	$\checkmark$	$\checkmark$	$\checkmark$	**0.999**	**1.686**	**0.976**	**1.716**	**1.158**
Model2	$\checkmark$	$\checkmark$	$\times$	0.998	1.777	0.974	1.798	1.191
Model3	$\checkmark$	$\times$	$\times$	0.998	1.807	0.973	1.921	1.379
Model4	$\checkmark$	$\times$	$\checkmark$	0.928	1.770	0.975	1.844	1.231

As shown in Table 2, the ablation results provide clear evidence of the respective contributions from LTPI and MPPGLI. The full TE-PINN (Model 1) achieves the best overall performance, yielding the lowest ubRMSE (1.686) and RMSE (1.716), while maintaining the highest Median $R^2$ (0.999). This indicates that the joint integration of LTPI and MPPGLI produces a synergistic effect, simultaneously enhancing predictive accuracy and physical consistency.

When LTPI is removed (Model 2), predictive performance declines moderately, with ubRMSE increasing to 1.777 and bias rising to 1.191. The absence of LTPI eliminates free-energy–based supervision, which weakens the alignment between latent state representations and thermodynamic dynamics. This reduction in internal regularization leads to a noticeable increase in systematic error. A more substantial degradation is observed when MPPGLI is removed (Model 3). Despite retaining LTPI, the lack of externally imposed physical constraints results in the largest RMSE (1.921) and bias (1.379) among the physics-informed variants. This demonstrates that MPPGLI plays a critical role in enforcing thermodynamic consistency, and its absence compromises predictive robustness, even though the overall $R^2$ remains relatively high (0.998). The baseline LSTM model (Model 4) performs the worst, with Median $R^2$ decreasing to 0.928. Although its ubRMSE (1.770) is slightly lower than that of Model 3, this apparent gain comes at the cost of reduced physical fidelity and generalizability, underscoring the limitations of purely data-driven approaches without physical priors.

Results confirm that both LTPI and MPPGLI are indispensable. In particular, the effect of these modules becomes more pronounced under challenging prediction scenarios. During periods of extreme temperature fluctuations, the absence of LTPI leads to larger prediction deviations, as the model loses the thermodynamic constraints that regulate latent energy evolution. This is especially evident in deeper soil layers, where temperature dynamics are governed by slower diffusion processes and require stronger physical regularization.Similarly, removing MPPGLI weakens the model’s ability to enforce diffusion-consistent temperature evolution over longer temporal horizons. Without the multi-pathway physical constraints, prediction errors tend to accumulate during multi-step forecasting, leading to larger biases in regions experiencing strong land–atmosphere coupling. LTPI stabilizes the latent thermodynamic space by embedding energy-based constraints, while MPPGLI ensures consistency with governing physical laws through multi-pathway regularization. Their joint integration substantially reduces both random and systematic errors, reinforcing the necessity of physics-guided architectures for accurate and physically consistent soil temperature prediction.

#### Loss weight-based sensitivity analysis

To quantitatively examine the interplay between physical regularization pathways and data-driven learning, we perform a sensitivity analysis on three critical hyperparameters (i.e., $\lambda _{phys}$, $\lambda _{pde}$, and $\lambda _{z}$), which control the relative weights of the thermodynamic consistency loss, the PDE-based residual loss, and the latent-variable supervision loss, respectively. As described in the Final Loss, the overall training objective combines data fidelity and multiple physics-guided regularization pathways through a normalized weighting mechanism. The coefficients $\lambda _{phys}$, $\lambda _{pde}$, and $\lambda _{z}$ therefore regulate the relative strength of thermodynamic constraints, PDE-based diffusion consistency, and latent-state supervision during optimization. To analyze the influence of different physics-guided constraints, we conduct a loss-weight sensitivity analysis under a normalized weighting scheme. The three coefficients satisfy the constraint $\lambda _{phys}+\lambda _{pde}+\lambda _{z}=1$. During the experiment, one coefficient is varied within the interval [0, 1], while the remaining two coefficients equally share the residual weight, i.e., $\lambda _j=\lambda _k=(1-\lambda _i)/2$. Under this design, the interval [0, 1] represents the full range of possible relative contributions of each physics-guided constraint. Smaller values correspond to weaker physical regularization (approaching purely data-driven learning), whereas larger values indicate stronger enforcement of the corresponding physical mechanism. This normalized exploration enables a systematic evaluation of how different physics-guided pathways influence model performance while maintaining a consistent overall regularization strength. The analysis spans three soil layers with increasing depth, including Level 1 (0-7 cm), Level 2 (7-28 cm), and Level 3 (28-100 cm). To ensure comparability, a controlled-variable strategy is adopted. When varying one hyperparameter, the other two are fixed at their baseline values, such that the observed changes in RMSE can be attributed solely to the target parameter.

**Figure 4:**
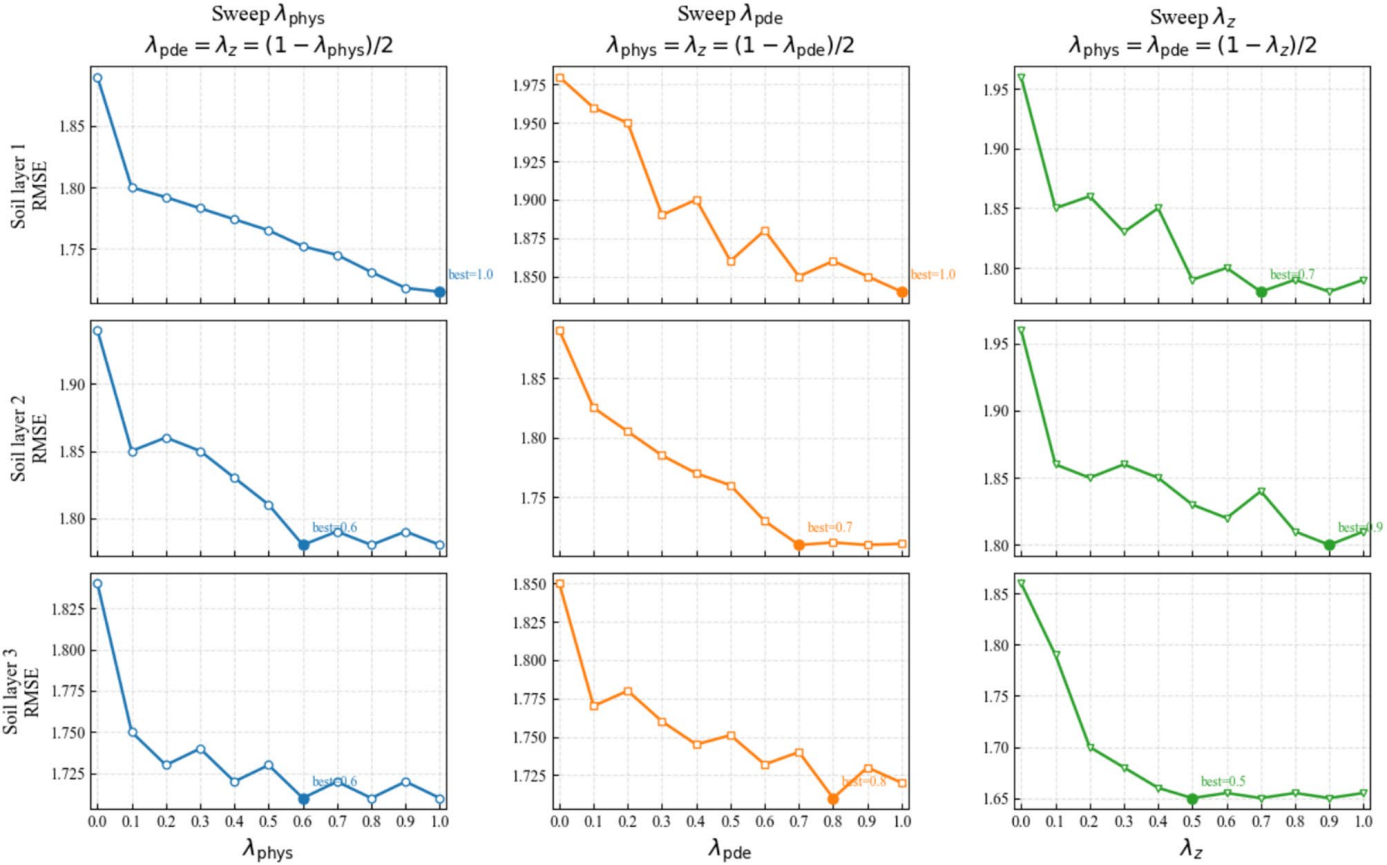
Impact of lambda weighting in the loss function across soil depths on soil temperature. In each experiment, one coefficient is varied from 0 to 1, while the remaining two coefficients equally share the residual weight so that $\lambda _{\textrm{phys}}+\lambda _{\textrm{pde}}+\lambda _z=1$. Rows correspond to soil layers, and columns correspond to the scans of $\lambda _{\textrm{phys}}$, $\lambda _{\textrm{pde}}$, and $\lambda _z$, respectively.

As shown in Fig. 4, the RMSE responses to $\lambda _{\textrm{phys}}$, $\lambda _{\textrm{pde}}$, and $\lambda _{z}$ exhibit a clear depth-dependent pattern, and the differences are evident both quantitatively and physically. For the shallow layer (Level 1, 0–7 cm), increasing $\lambda _{\textrm{phys}}$ yields the most regular and persistent improvement, with RMSE decreasing almost monotonically from about 1.88 at $\lambda _{\textrm{phys}}=0$ to about 1.71 at $\lambda _{\textrm{phys}}=1.0$. By comparison, the sweep of $\lambda _{\textrm{pde}}$ reduces RMSE more modestly, from about 1.98 to about 1.85, and the curve shows noticeable local fluctuations around $\lambda _{\textrm{pde}}=0.2$–0.8. The $\lambda _{z}$ pathway produces an intermediate behavior, lowering RMSE from about 1.96 to about 1.79, but still with visible oscillations. This indicates that near-surface prediction is most sensitive to thermodynamic balance and energy-dissipation regulation, whereas purely diffusion-based constraints are less effective because the shallow soil layer is more strongly perturbed by short-term atmospheric forcing and surface flux variability. For the intermediate layer (Level 2, 7–28 cm), the dominant role gradually shifts from $\lambda _{\textrm{phys}}$ to $\lambda _{\textrm{pde}}$. The $\lambda _{\textrm{phys}}$ curve decreases from about 1.93 to about 1.78, but the reduction is not strictly monotonic and slight rebounds appear after $\lambda _{\textrm{phys}}\approx 0.6$. In contrast, increasing $\lambda _{\textrm{pde}}$ produces a clearer and more sustained improvement, with RMSE dropping from about 1.89 to about 1.71 and becoming nearly flat after $\lambda _{\textrm{pde}}\approx 0.8$, indicating a more stable optimum. The effect of $\lambda _{z}$ is also positive, reducing RMSE from about 1.96 to about 1.80, but its improvement remains weaker than that of $\lambda _{\textrm{pde}}$. This behavior is physically consistent with the transitional role of the intermediate soil layer, where the direct impact of surface forcing is attenuated and vertical heat diffusion becomes more dominant, making PDE-based constraints more effective. For the deeper layer (Level 3, 28–100 cm), the strongest sensitivity is observed for $\lambda _{z}$. As $\lambda _{z}$ increases, RMSE drops sharply from about 1.86 at $\lambda _{z}=0$ to about 1.70 at $\lambda _{z}=0.2$, and further decreases to about 1.65 around $\lambda _{z}=0.5$–0.9, which is clearly lower than the minima obtained by the other two pathways. By contrast, the $\lambda _{\textrm{phys}}$ sweep reduces RMSE from about 1.83 to about 1.71, and the $\lambda _{\textrm{pde}}$ sweep decreases it from about 1.85 to about 1.71, both showing weaker overall gains than $\lambda _{z}$. This suggests that, in deeper horizons, latent-state regularization becomes most important because the system evolves more slowly, exhibits stronger thermal memory, and is less directly controlled by observable surface forcing. Under such conditions, a well-regularized latent thermodynamic state is more effective in capturing the hidden persistence and cumulative diffusion behavior of subsurface temperature dynamics.

Overall, results confirm that the contributions of different physics-informed losses are strongly depth-dependent. $\lambda _{phys}$ is most beneficial near the surface (0-7 cm), $\lambda _{pde}$ dominates in the intermediate subsurface (7-28 cm), and $\lambda _z$ is crucial for deeper horizons (28-100 cm). This depth-specific sensitivity suggests that no single constraint is universally optimal across soil layers. Adaptive weighting of physical priors is necessary to maximize predictive skill. This finding is consistent with recent advances in hydrological modeling, where depth-aware constraints improve robustness and generalization. These observations confirm that the weighting parameters play a critical role in balancing different physical pathways and provide empirical guidance for selecting stable and physically consistent configurations.

### Temporal robustness evaluation

**Figure 5:**
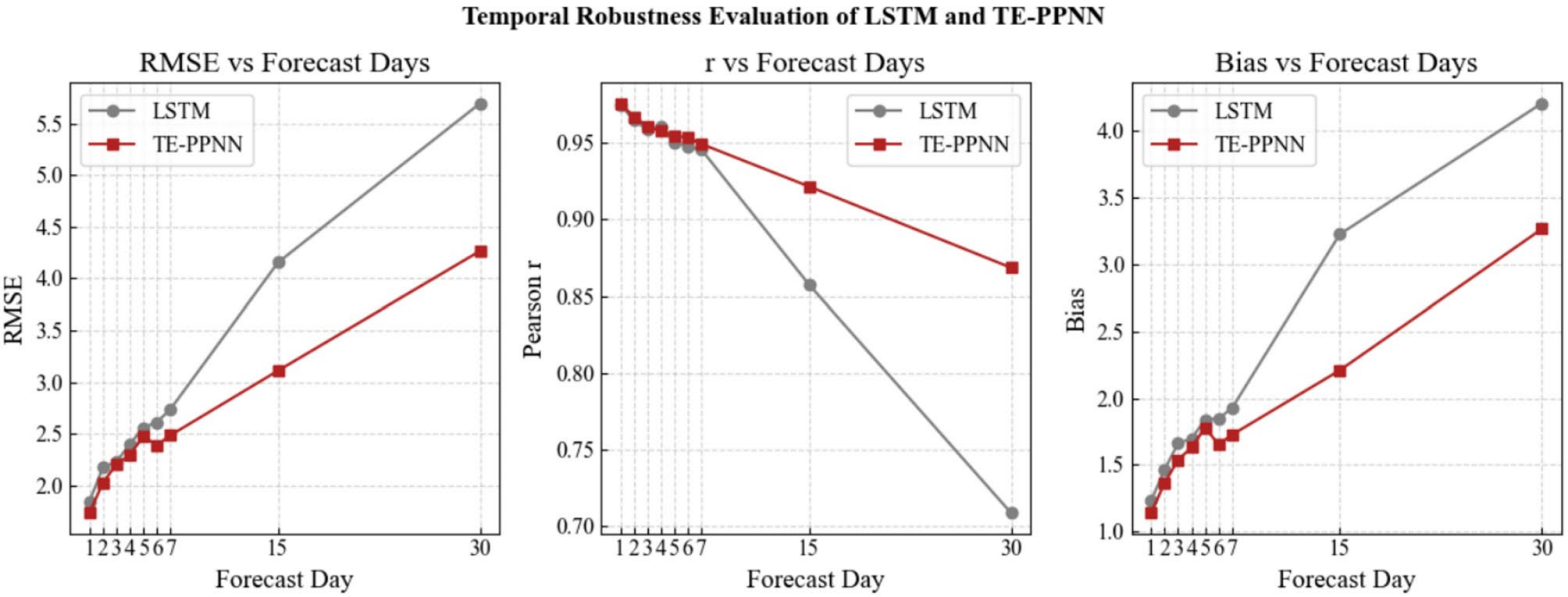
Comparison of RMSE, correlation coefficient(*r*), and bias between LSTM and TE-PINN over 1–30 forecast days. TE-PINN consistently achieves lower RMSE and bias, and higher *r*, with significantly improved stability over long lead times.

We examine the generalization of TE-PINN in long-term forecasting. Periodic experiments are performed, and results are compared with the LSTM baseline. The evaluation employs three widely accepted metrics–RMSE, Pearson correlation coefficient (*r*), and bias as illustrated in Fig. 5. Comparison of RMSE, *r*, and bias between LSTM and TE-PINN over short- and extended-range forecast horizons (1–7, 15, and 30 days) TE-PINN consistently achieves lower RMSE and bias, and higher *r*, with significantly improved stability over long lead times.

As the forecast horizon increases, both models experience a gradual decline in performance, which is expected due to error propagation and the accumulation of uncertainty over time. Nevertheless, TE-PINN consistently outperforms LSTM across all lead times, with its advantage becoming particularly evident in long-term forecasts. As shown in Fig. 5, the predictive performance of both models gradually declines as the forecast horizon increases, which is expected because autoregressive errors accumulate during iterative rollouts. However, the degradation of TE-PINN is consistently slower than that of LSTM. From Day 3 to Day 7, the RMSE of TE-PINN increases only moderately from 1.83 to 2.08, whereas LSTM rises from 1.94 to 2.74. When the prediction horizon is extended to 15 and 30 days, the gap becomes even more pronounced. This trend suggests that the proposed physics-guided framework provides stronger trajectory-level stability in long-range prediction. In conventional LSTM forecasting, small one-step errors may propagate through hidden states and gradually lead to trajectory drift. By contrast, TE-PINN constrains the latent evolution through thermodynamic admissibility and diffusion-consistency regularization, which suppresses physically implausible drift and preserves more realistic soil temperature dynamics over extended lead times. This demonstrates that TE-PINN retains predictive accuracy more effectively over extended horizons. TE-PINN maintains higher correlation values, declining only slightly from 0.9607 to 0.9494 between Days 3 and 7, while LSTM shows a steeper reduction from 0.9623 to 0.9437. This indicates that TE-PINN preserves a stronger alignment between predicted and observed soil temperatures, even under extended forecast conditions. Even at the 30-day horizon, TE-PINN retains a correlation coefficient of 0.87, whereas LSTM drops to 0.71, indicating stronger temporal consistency in long-range predictions. The bias metric further highlights the superiority of TE-PINN. While both models exhibit an increase in systematic error with longer lead times, TE-PINN consistently produces lower bias values. The difference is particularly evident in late-stage forecasts (Days 6-7), where LSTM tends to overestimate soil temperature, whereas TE-PINN mitigates this error by incorporating thermodynamic priors and physics-based constraints.

**Figure 6:**
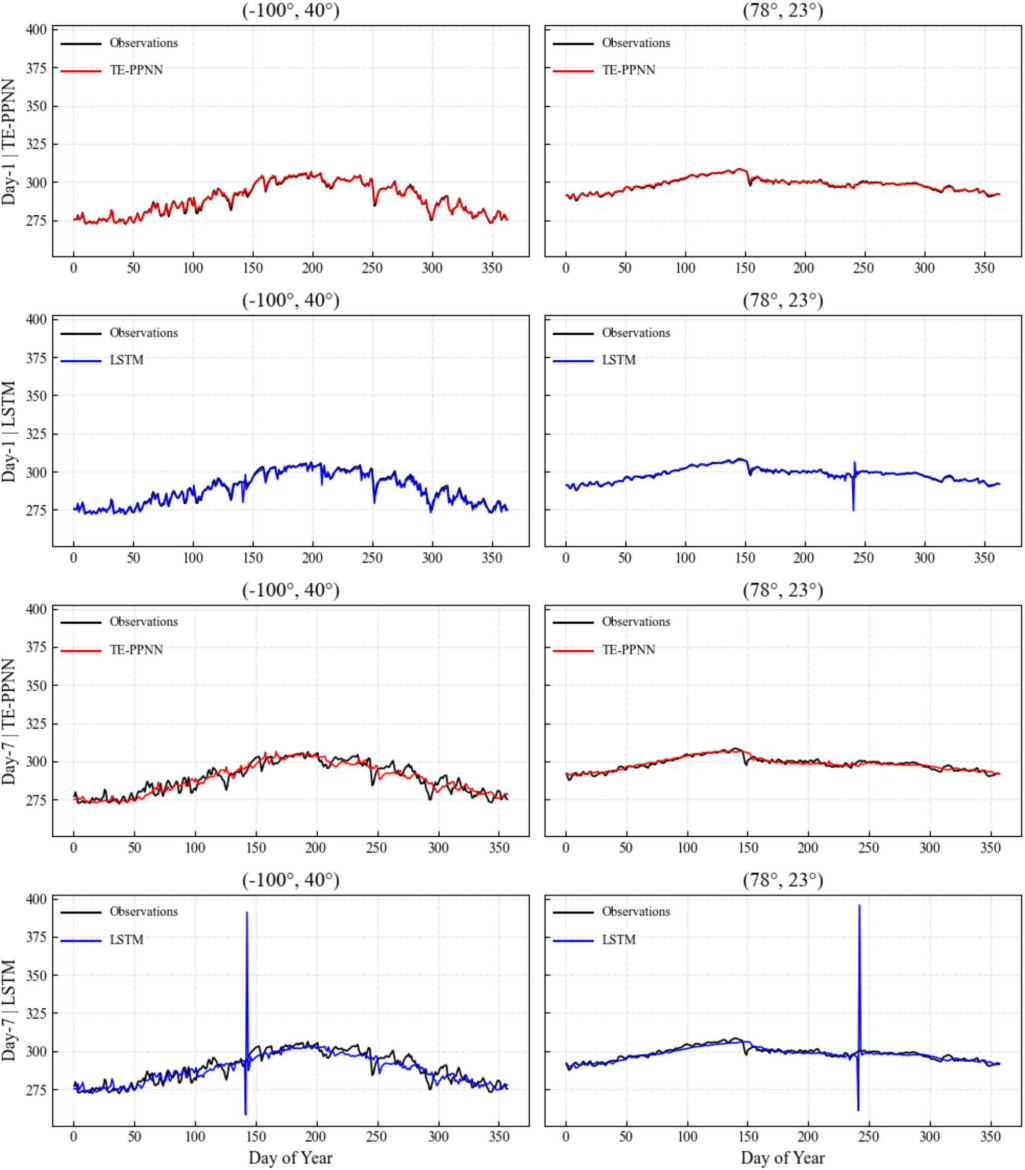
Comparison of observed soil temperature with predictions from TE-PINN (red) and LSTM (blue) at two representative sites: ($-100^\circ$ , $40^\circ$ ) in central North America (continental agricultural region) and ($78^\circ$ , $23^\circ$ ) in central India (monsoon climate zone). Results are presented across the full annual cycle (x-axis: day of year 1–365; y-axis: soil temperature in K) for forecasts with daily periodicity (1-day lead) and weekly periodicity (7-day lead).

Fig. 6 complements these results by comparing annual soil temperature cycles at representative sites. TE-PINN predictions (red) closely follow observed trends with smooth trajectories, whereas LSTM (blue) exhibits strong noise and spurious fluctuations, particularly under 7-day lead forecasts. These spikes mainly arise from error accumulation during autoregressive prediction when no physical constraints are imposed. Without diffusion-based regularization, the LSTM model may generate temporally inconsistent temperature trajectories, leading to unrealistic fluctuations under rapidly changing surface forcing. For long-term forecasts over central India, noise is particularly noticeable in the LSTM prediction trend, resulting in significantly greater volatility compared to short-term forecasts. In contrast, the TE-PINN exhibits strong forecast robustness, resulting in smaller long-term forecast errors. The result indirectly demonstrates the benefit of LTPI and MPPGLI. By enforcing physical constraints, they significantly enhance long-term forecasts and bring predicted trends closer to observations. Meanwhile, these oscillations reflect LSTM’s reliance on statistical correlations without physical regularization, leading to unstable predictions. By contrast, TE-PINN embeds latent thermodynamic regulation and PDE-based constraints, which suppress random noise and stabilize long-horizon forecasts.

Overall, the temporal robustness analysis shows that TE-PINN delays error accumulation. It also maintains stronger predictive consistency than purely data-driven methods. By constraining uncertainty propagation and embedding latent thermodynamic regulation, TE-PINN offers a more reliable and physically consistent framework for long-horizon soil temperature forecasting.

**Figure 7:**
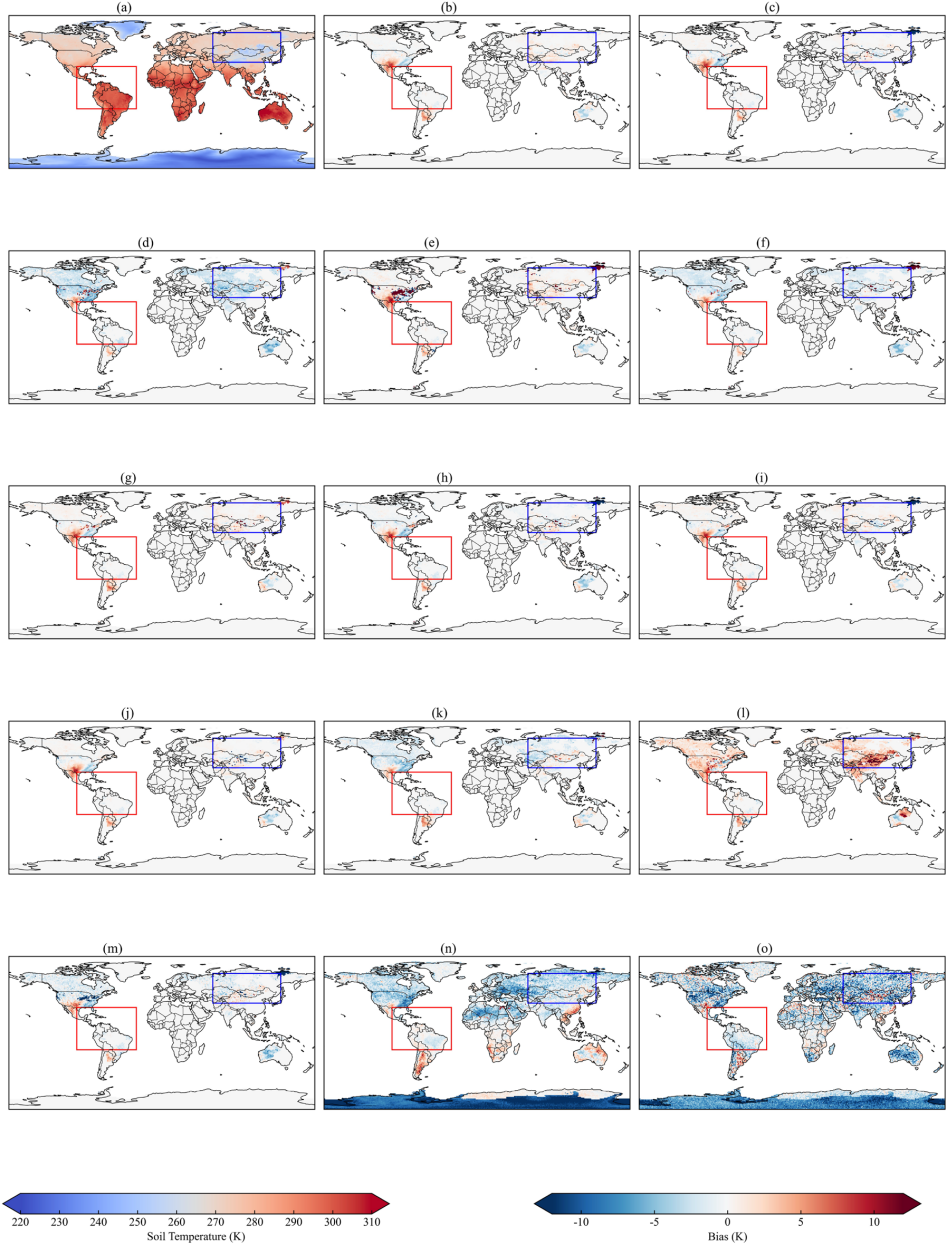
Global soil temperature patterns and prediction bias distributions. Panel (a) shows the observed soil temperature field, while panels (b–o) display the prediction bias (prediction - observation) for TE-PINN and baseline models. Red and blue rectangles highlight representative regions with pronounced prediction bias. The left color represents absolute soil temperature (K), whereas the right color indicates prediction bias (K).

### Comparison with state-of-the-art models

To comprehensively evaluate the performance of the proposed TE-PINN model, we consider two complementary benchmark groups corresponding to the two baseline categories reported in Tables 3 and 4: (i) shallow recurrent baselines, mainly composed of LSTM-based variants that capture temporal correlations but lack explicit physical constraints, and (ii) deep baseline architectures, which include more complex sequence modeling frameworks commonly used in modern meteorological forecasting tasks. As shown in Table 3, from the shallow-baseline perspective, we compare TE-PINN with LSTM-type architectures, including LSTM^48^, AttentionLSTM^49^, EDLSTM^50^, AEDLSTM^51^, LSTM-ML^52^, FB-LSTM^53^, and FAMLSTM^54^. These models mainly capture temporal correlations and single-path physical relationships and rely on limited recurrent feature representations. As shown in Table 4, from the deep-baseline perspective, we compare TE-PINN with several representative sequence modeling architectures that have recently been applied in meteorological and environmental prediction tasks. These models are selected because they capture different types of temporal and spatiotemporal dependencies that are relevant for soil temperature dynamics. Trans-LSTM combines transformer-style attention with recurrent structures to enhance long-range temporal representation, which is important for modeling the delayed response of soil temperature to atmospheric forcing^55^. MD-Transformer introduces multi-scale attention mechanisms that can capture hierarchical temporal patterns in meteorological variables such as radiation and soil moisture^56^. Skip-Transformer employs skip connections to stabilize long-sequence learning and mitigate gradient degradation during extended autoregressive forecasting^57^. MMamba represents a state-space sequence model designed to efficiently capture long-range dependencies with reduced computational cost, making it suitable for large-scale environmental datasets^58^. In addition, XGBoost is included as a strong ensemble baseline widely used in environmental prediction tasks^59^. Although it is not a deep neural network model, it is frequently used as a competitive machine-learning benchmark in geoscientific forecasting studies. Together, these baselines cover diverse modeling paradigms for handling long-term temporal dependencies and complex environmental forcing, providing a comprehensive comparison for evaluating the effectiveness of the proposed physics-guided framework.

As shown in Table 3, the shallow baselines exhibit notable limitations in predictive stability and physical fidelity. To further examine whether more sophisticated feature interaction mechanisms could enhance the fusion of static and dynamic inputs, we implemented a TE-PINN variant augmented with an attention module. The attention layer was inserted after the input concatenation stage to dynamically reweight temporal features. As reported in Table3, the TE-PINN + Attention variant achieves a median $R^2$ of 0.898, ubRMSE of 1.810, correlation coefficient *R* of 0.873, RMSE of 2.013, and bias of 1.492, which are all inferior to those of the original TE-PINN model. To further investigate whether the performance improvement stems from physical learning rather than simply stronger regularization, we compared TE-PINN with several LSTM-based architectures (such as Attention-LSTM and EDLSTM), which already incorporate implicit regularization through their architectural design. Despite this built-in regularization, TE-PINN consistently achieves lower prediction errors. In addition, the sensitivity analysis in Fig. 4 shows that the model performance varies systematically with the strength of the physics-guided loss terms. These observations suggest that the physics-guided loss acts as a structured inductive bias grounded in soil thermal diffusion physics, rather than as a purely statistical regularizer. Conventional recurrent models such as LSTM and RNN maintain relatively high correlation coefficients (*R* = 0.97) but suffer from large RMSE and bias values, indicating unstable temporal representations under complex climatic variability. AttentionLSTM and FB-LSTM, which introduce attention or feedback mechanisms, provide only marginal gains and show poor generalization in regions with strong land–atmosphere coupling. Physics-augmented models such as EDLSTM, AEDLSTM, and FAMLSTM achieve higher $R^2$ values (> 0.97) but still yield elevated RMSE (up to 2.57) and bias (up to 2.53), suggesting that single-pathway physical constraints cannot guarantee thermodynamic consistency. Note that Fig. 7 presents both the observed temperature field and the spatial distribution of prediction bias to illustrate the regional performance of different models. Panel (a) shows the observed soil temperature distribution, while panels (b–o) display the prediction bias (prediction - observation) for TE-PINN and the baseline models. Compared with the recurrent baselines, TE-PINN exhibits substantially smaller bias magnitudes and smoother spatial error structures, indicating improved consistency with the observed large-scale temperature gradients. In contrast, conventional recurrent architectures such as LSTM and RNN show clear systematic warm biases in several regions, particularly within the highlighted boxes. More complex deep sequence models (e.g., Trans-LSTM, MD-Transformer, Skip-Timeformer, and MMamba) exhibit even stronger spatial noise and fragmented bias patterns. This degradation likely arises from their heavy reliance on purely data-driven spatiotemporal correlations, which can amplify regional prediction errors when physical constraints are absent. These discrepancies are especially visible in high-latitude regions and areas with complex land–atmosphere interactions, where the absence of physically guided diffusion constraints leads to unstable spatial error structures. In contrast, the thermodynamically guided formulation of TE-PINN(panel b) produces more coherent spatial predictions and substantially reduces large-scale bias accumulation. These results highlight the importance of incorporating physically guided constraints for achieving stable spatial predictions in long-term soil temperature forecasting.

As presented in Table 4, the deep baseline methods demonstrate enhanced feature extraction capacity but still suffer from physical inconsistency and regional bias. Trans-LSTM and MD-Transformer achieve high $R^2$ values (0.996 and 0.993), indicating strong temporal learning ability. However, their RMSE and bias (2.33–3.06 and 1.92–2.39) are substantially higher than those of TE-PINN. Skip-Timeformer and MMamba partially improve long-sequence modeling through skip connections and multimodal attention, yet their physical interpretability remains limited, resulting in unstable bias across regions with strong land–atmosphere coupling. XGBoost, as a non-deep ensemble approach, further highlights the gap between data-driven prediction and physical realism, exhibiting the largest RMSE (4.996) and bias (4.056). To better understand this discrepancy, it is important to note that these deep baseline architectures represent several major paradigms of modern sequence modeling commonly applied in environmental forecasting. Trans-LSTM integrates attention mechanisms with recurrent structures to capture long-range temporal dependencies, while MD-Transformer and Skip-Timeformer rely on transformer-based attention mechanisms designed to learn multi-scale temporal patterns from meteorological forcing variables. MMamba belongs to the family of state-space sequence models that efficiently capture long temporal contexts with reduced computational cost. These models therefore provide strong reference baselines for evaluating the predictive capability of the proposed TE-PINN framework.

Despite their strong sequence modeling capability, these architectures remain purely data-driven and do not explicitly enforce the governing physics of soil heat transfer. Soil temperature evolution is primarily controlled by diffusion-dominated heat propagation and thermal inertia within the soil column, which leads to smooth temporal dynamics and gradual vertical propagation of temperature signals. When such physical constraints are absent, purely data-driven autoregressive forecasting models may accumulate small prediction errors within the hidden state, which can gradually amplify over time. This phenomenon becomes particularly evident when the model extrapolates beyond the training distribution or encounters rapidly varying surface forcing. As a result, the predicted trajectories may exhibit unrealistic spikes or abrupt fluctuations, as observed in Fig. 6 for the non–physics-informed LSTM model.

In contrast, TE-PINN embeds physically meaningful inductive biases directly into the learning process. The thermodynamic consistency constraint regulates the evolution of latent energy states, ensuring that predicted temperature changes remain physically admissible. Meanwhile, the PDE-based diffusion residual explicitly enforces smooth vertical heat propagation along the soil column, which is the dominant physical mechanism governing soil temperature dynamics. In addition, the latent-state supervision pathway stabilizes hidden-state evolution during autoregressive forecasting. These physics-guided mechanisms jointly restrict the model from generating temporally inconsistent trajectories and reduce the amplification of prediction errors over long forecasting horizons.

Consequently, TE-PINN achieves systematic improvements across all evaluation metrics reported in Table 4. For example, the RMSE of TE-PINN (1.716) is substantially lower than that of Trans-LSTM (2.425) and MD-Transformer (3.060), while the prediction bias is reduced from 1.919–2.388 to 1.158. At the same time, TE-PINN attains the highest correlation coefficient ($R=0.976$) and median $R^2$ value (0.999), indicating stronger predictive consistency across spatial locations. These results suggest that the advantage of TE-PINN does not primarily stem from increased architectural complexity, but rather from incorporating physically consistent constraints that better reflect the governing processes of soil temperature dynamics.

In addition to predictive accuracy, computational efficiency is also an important consideration for practical deployment. The computational characteristics of the proposed TE-PINN framework are summarized in Table 3 and Table 4. Compared with several deep baseline models such as MD-Transformer, Skip-Timeformer, and MMamba, the proposed framework maintains a relatively lightweight computational footprint in terms of both FLOPs and parameter size. Unlike transformer-based architectures that often introduce substantial parameter growth through stacked attention layers, TE-PINN incorporates thermodynamic and diffusion-based constraints through auxiliary loss terms rather than additional trainable modules. As a result, the physics-guided formulation mainly affects the training stage while maintaining comparable inference complexity to the underlying LSTM-based structure. This design enables TE-PINN to improve physical consistency and long-range predictive stability without introducing substantial computational overhead.

Overall, the evaluation results indicate that among all assessed models, TE-PINN achieves the best balance between predictive accuracy and physical rationality. Unlike recurrent or transformer-based architectures that rely solely on statistical dependencies, TE-PINN explicitly embeds thermodynamic priors and partial differential equation residuals into the learning process. This integration enables the model to maintain stability under nonlinear boundary forcing and to ensure energy-conservative predictions across different climatic regions. The latent thermodynamic inference in TE-PINN alleviates the bias–variance imbalance observed in other models, providing physically consistent predictions that closely align with observed soil temperature dynamics. These advantages make TE-PINN a robust and effective predictive framework for meteorological applications.

**Table 3:** Performance comparison between TE-PINN and shallow baseline methods.

Model	FLOPs (G)	Params (M)	Median $R^{2}$	ubRMSE	*R*	RMSE	Bias
LSTM	0.08	0.20	0.893	1.737	0.975	1.749	1.192
RNN	0.02	0.05	0.993	2.024	0.968	2.992	2.534
AttentionLSTM	0.10	0.24	0.656	1.843	0.973	1.871	1.323
EDLSTM	0.15	0.40	0.974	1.769	0.975	2.572	1.224
AEDLSTM	0.17	0.44	0.997	1.733	0.976	1.813	1.233
LSTM-ML	0.09	0.23	0.890	1.760	0.975	1.803	1.231
FB-LSTM	0.09	0.22	0.589	1.765	0.965	1.805	1.247
FAM-LSTM	0.11	0.26	0.984	1.732	0.975	1.750	1.175
TE-PINN + Attention	0.18	0.38	0.898	1.810	0.873	2.013	1.492
**TE-PINN**	0.16	0.34	**0.999**	**1.686**	**0.976**	**1.716**	**1.158**

**Table 4:** Performance comparison between TE-PINN and deep baseline methods.

Model	FLOPs (G)	Params (M)	Median $R^2$	ubRMSE	*R*	RMSE	Bias
Trans-LSTM	0.42	0.82	0.996	2.332	0.967	2.425	1.919
MD-Transformer	0.96	1.94	0.993	2.537	0.948	3.060	2.388
Skip-Timeformer	0.88	1.71	0.858	2.171	0.961	2.609	1.995
MMamba	0.73	1.36	0.966	2.540	0.947	2.814	2.263
XGBoost	0.01	0.08	0.956	3.999	0.846	4.996	4.056
**TE-PINN**	0.16	0.34	**0.999**	**1.686**	**0.976**	**1.716**	**1.158**

## Discussion

Despite substantial progress in data-driven soil temperature forecasting, several critical challenges remain unresolved. First, most existing methods treat soil temperature purely as a statistical time series, ignoring the intrinsic thermodynamic constraints that govern energy transfer in soil systems. This often leads to predictions that violate basic physical principles, especially under data scarcity or long-term forecasting. Second, conventional architectures lack explicit mechanisms to handle multiscale temporal dependencies and heterogeneous spatial conditions, resulting in degraded generalization across diverse climatic and land cover regimes. Third, the interpretability of deep learning models remains limited, hindering their adoption in operational earth system monitoring where physical consistency and transparency are paramount.

A major limitation of conventional recurrent architectures such as LSTM, RNN, and attention-based hybrids is their reliance on statistical dependencies in low-dimensional feature space. These models struggle to represent latent thermodynamic states and cannot enforce energy conservation or dissipation constraints, which are essential for long-term stability. In contrast, TE-PINN introduces a latent thermodynamic potential inference module that learns internal variables *z* and their temporal evolution $\dot{z}$, from which a free energy sequence $\psi$ is inferred. This free energy representation provides a latent state regularization pathway, ensuring that model predictions remain bounded within thermodynamically feasible trajectories, even in the presence of noisy or sparse input.

Moreover, the model explicitly computes free energy differences and their spatial derivatives to quantify internal forces $\tau$, from which dissipation rates *D* are derived. These quantities are incorporated into loss functions such as $L_{diss}$ and $L_{energy}$, forming a physically grounded supervision mechanism. By enforcing dissipation consistency and energy balance, the model suppresses unphysical oscillations and stabilizes the forecast trajectory over long horizons. The ablation study demonstrates that excluding physics-informed losses significantly degrades generalization accuracy in long-range forecasts, emphasizing their indispensable contribution. Another important advantage of TE-PINN lies in its MPPGLI. Beyond the latent free energy constraints, the model introduces a PDE residual loss $L_{pde}$ derived from the one-dimensional heat conduction equation, which is computed using the temporal derivative $\partial T/\partial t$ and the spatial second derivative $\partial ^2T/\partial z^{2\ }$from different soil layers (T1-T3). This loss enforces external consistency with the governing equations of heat transport. Combining latent regulation with PDE enforcement yields a synergistic effect. Sensitivity and layer-wise analyses show that slow diffusion layers benefit most, enhancing long-term stability and convergence.

Although the proposed framework demonstrates strong predictive performance and physical consistency, its thermodynamic formulation primarily targets diffusion-dominated soil temperature dynamics under typical environmental conditions. In situations characterized by strong external disturbances, such as intense precipitation events or rapid surface energy fluctuations, soil thermal processes may temporarily deviate from quasi-equilibrium assumptions. Under these conditions, additional coupled processes such as rapid moisture redistribution and nonlinear heat–water interactions may become more prominent. While the current formulation captures the dominant vertical diffusion dynamics, incorporating more detailed multiphysics representations of soil heat–moisture coupling could further improve model robustness under highly transient environmental forcing. This provides a promising direction for future extensions of the proposed framework.

Overall, TE-PINN demonstrates a unique ability to couple latent thermodynamic state regulation with external physical balance enforcement, forming a robust hybrid architecture. This design mitigates error accumulation, enhances temporal robustness, and improves spatial transferability. The framework systematically addresses several limitations in current soil temperature forecasting. Physics-based constraints reduce reliance on statistical correlations. Thermodynamic plausibility is enforced through free-energy and dissipation modeling. Governing PDEs anchor predictions within physical laws. Static site features and auxiliary variable supervision further enhance interpretability. Collectively, these components yield higher predictive accuracy, reduced systematic drift, and improved stability over extended forecasting horizons, positioning TE-PINN as a physically consistent and reliable paradigm for soil temperature prediction.

## Conclusion

In this study, we propose the TE-PINN framework to address the limitations of existing soil temperature prediction methods in long-term forecasting and spatial generalization. By embedding physical constraints derived from free-energy theory and thermodynamic dissipation into the model design, TE-PINN achieves a robust integration of physics and deep learning that advances beyond conventional data-driven approaches. The framework is built upon LSTM encoders and incorporates the LTPI and MPPGLI module. The LTPI module infers latent thermodynamic states and their temporal dynamics through free-energy computation, energy difference estimation, and dissipation quantification, thereby constraining forecasts within thermodynamically feasible bounds. The MPPGLI module combines multiple physics-guided loss functions, including PDE residuals based on heat conduction, dissipation derived from entropy production, and energy balance guided by the Clausius–Duhem inequality. These pathways enforce external consistency with physical laws while complementing internal latent regulation. Their synergy enhances long-range stability, reduces error accumulation, and strengthens robustness under heterogeneous land–atmosphere interactions. Experimental evaluations on multi-year, multi-layer soil temperature datasets show that TE-PINN consistently outperforms shallow and deep baseline methods. These results confirm its predictive accuracy and generalization capability across different climatic regions.

Despite these improvements, TE-PINN introduces additional physical loss terms and optimization procedures that increase computational costs compared with traditional models. Future research should focus on reducing training overhead while maintaining physical interpretability. Promising directions include the use of variational inference for efficient uncertainty quantification, the development of surrogate models to accelerate physics-guided learning, and the application of knowledge distillation to transfer physical constraints into lightweight architectures. Advancing along these directions may enhance the practicality of TE-PINN for large-scale and real-time soil temperature forecasting.

## Data Availability

The data used in this study are derived from the LandBench 1.0 dataset, which is publicly available and described in Li et al. (2024) (https://doi.org/10.1016/j.eswa.2023.122917). The dataset and its associated toolbox can be accessed via: https://github.com/2023ATAI/Landbench1.0. LandBench 1.0 integrates multiple widely used and publicly accessible geophysical datasets, including: (i) ERA5 and ERA5-Land reanalysis data provided by the European Centre for Medium-Range Weather Forecasts (ECMWF) (https://www.ecmwf.int/), (ii) SoilGrids data provided by ISRIC - World Soil Information (https: //www.isric.org/), (iii) MODIS satellite products provided by NASA (https://modis.gsfc.nasa.gov/). These datasets are well-established, quality-controlled, and widely used in environmental and climate research, ensuring the reliability and validity of the data used in this study.

## Code availability

The code and scripts developed for this study have been archived in a DOI-minting repository (Zenodo) and are publicly available at: https://doi.org/10.5281/zenodo.19383807. This archived version ensures long-term accessibility and reproducibility of the computational framework.
